# Shared gray matter alterations in individuals with diverse behavioral addictions: A voxel-wise meta-analysis

**DOI:** 10.1556/2006.2020.00006

**Published:** 2020-04-07

**Authors:** Kun Qin, Feifei Zhang, Taolin Chen, Lei Li, Wenbin Li, Xueling Suo, Du Lei, Graham J. Kemp, Qiyong Gong

**Affiliations:** 1 Huaxi MR Research Center (HMRRC), Department of Radiology, West China Hospital of Sichuan University, Chengdu, Sichuan, China; 2 Psychoradiology Research Unit of Chinese Academy of Medical Sciences, West China Hospital of Sichuan University, Chengdu, Sichuan, China; 3 Department of Psychiatry and Behavioral Neuroscience, University of Cincinnati College of Medicine, Cincinnati, OH, USA; 4 Liverpool Magnetic Resonance Imaging Centre (LiMRIC), Institute of Ageing and Chronic Disease, University of Liverpool, Liverpool, UK

**Keywords:** behavioral addictions, magnetic resonance imaging, gray matter, voxel based morphometry, meta-analysis

## Abstract

**Background and aims:**

Numerous studies on behavioral addictions (BAs) have reported gray matter (GM) alterations in multiple brain regions by using voxel-based morphometry (VBM). However, findings are poorly replicated and it remains elusive whether distinct addictive behaviors are underpinned by shared abnormalities. In this meta-analysis, we integrated VBM studies on different BAs to investigate common GM abnormalities in individuals with BAs.

**Methods:**

We performed a systematic search up to January 2019 in several databases for VBM studies investigating GM differences between individuals with BAs and healthy controls. The reference lists of included studies and high-quality reviews were investigated manually. Anisotropic effect-size signed differential mapping was applied in this meta-analysis.

**Results:**

Twenty studies including 505 individuals with BAs and 564 healthy controls met the inclusion criteria. Compared with healthy controls, individuals with BAs showed GM atrophy in the left anterior cingulate (extending to the left medial superior frontal gyrus and bilateral orbitofrontal gyrus), right putamen and right supplementary motor area. Subgroup analysis found heterogeneity in gender and subtypes of BAs. Meta-regression revealed that GM decreases in the left anterior cingulate and right supplementary motor area were positively correlated with addictive severity. Higher impulsivity was associated with smaller volume of the left anterior cingulate.

**Discussion and conclusions:**

Our findings on BAs were mainly derived from internet gaming disorder (IGD) and pathological gambling (PG) studies, preliminarily suggesting that GM atrophy in the prefrontal and striatal areas might be a common structural biomarker of BAs.

## Introduction

Behavioral addictions (BAs), also known as non-substance addictions, are a constellation of recognizable and clinically significant syndromes characterized by distress or interference with personal functions that develop as a result of repetitive rewarding behaviors other than the use of dependence-producing substances (World Health Organization, 2018). Indulgence towards addictive activities is no longer rare in recent years. The estimated 12-month prevalence of BAs in U.S. adults is between 2% (Internet addiction) and 10% (work addiction), turning addiction especially BAs into a growing mental health issue (Sussman, Lisha, & Griffiths, 2011). Various as addictive behaviors are, individuals with BAs share chronic manifestations including craving, tolerance, impulsiveness and withdrawal symptoms, which ultimately result in a constellation of adverse consequences, such as financial difficulties, incarceration, family disharmony, and impaired social relationships (Yau & Potenza, 2015).

BAs were first acknowledged since the reclassification of pathological gambling (PG) as non-substance addictive disorder in the fifth edition of the Diagnostic and Statistical Manual of Mental Disorders (DSM-5) (American Psychiatric Association, 2013). Moreover, Recent inclusion of internet gaming disorder (IGD) in the eleventh revision of International Classification of Diseases (ICD-11) suggests the growing influence of BAs. A number of brain researches have demonstrated the underlying neural correlates of IGD and PG, further reinforcing the concept of BAs as psychiatric disorders. Though insufficient evidences and criteria for definition of other types of BAs, some leading studies have extended the field of BAs beyond gambling to include different behaviors encompassing internet gaming, exercise, working, shopping, eating, social media, and sex (Baxter, Craig, Cotton, & Liney, 2019; Hausenblas, Schreiber, & Smoliga, 2017; Lior, Abira, & Aviv, 2018; Petry, Zajac, & Ginley, 2018).

From the perspective of IGD and PG as prototypical disorders, there are substantial similarities between BAs and substance addictions in comorbidities, diagnostic criteria, cognitive features, and neural correlates (Fauth-Buhler, Mann, & Potenza, 2017). Impaired reward system and subsequent reinforcement learning can obviously illustrate the common pathway of both. However, such abnormalities derived from behavior itself without the neurotoxic effect of drugs are inexplicable, indicating subtle dissimilarities between the neural mechanism of BAs and substance addictions (Robbins & Clark, 2015). Fortunately, the absence of drug effects benefits the discovery of real psychopathology in BAs, which is almost impossible in substance addiction. Moreover, BAs have been reported to be associated with gender-related vulnerability. For example, individuals with IGD and PG are generally males, while most compulsive buyers are females (Dong, Wang, Du, & Potenza, 2018; Maraz, Griffiths, & Demetrovics, 2016). Considering these distinct features from substance addiction, investigation of neurobiological abnormalities in BAs may provide diagnostically and therapeutically useful insight into this novel mental disorder.

With the development of high-resolution magnetic resonance imaging (MRI) and advanced image-analytic techniques, brain structural and functional abnormalities can be readily detected and localized. As an automated quantitative method for morphological analysis, voxel-based morphometry (VBM) has been widely applied in mental disorders to find evidence of gray matter (GM) alterations between patients and healthy control subjects (Ashburner & Friston, 2001). Different addictive behaviors as various BAs are involved in, relevant VBM studies have reported altered GM volume mainly in prefrontal and striatal regions. Specifically, a systematic review on neuroimaging studies of IGD have shown structural abnormalities and resting-state dysfunction within the prefrontal-striatal circuits (Weinstein, 2017). Similar findings have also been identified in pathological gamblers, with higher volume of prefrontal cortex and ventral striatum and increased functional connectivity between them (Koehler, Hasselmann, Wustenberg, Heinz, & Romanczuk-Seiferth, 2015; Koehler et al., 2013). As for food addiction, compulsive sexual behavior and internet communication addiction, structural and functional results can also be partially replicated in the prefrontal or striatal areas (Contreras-Rodriguez, Martin-Perez, Vilar-Lopez, & Verdejo-Garcia, 2017; Montag & Zhao, 2018; Schmidt et al., 2017). However, inconsistency still exists, and no robust conclusions can be obtained. For example, GM volume in precentral gyrus was increased in a study of problematic hypersexual behavior (Seok & Sohn, 2018b), but the opposite was found in IGD individuals (Sun et al., 2014), while two studies of PG found no significant GM alterations (van Holst, de Ruiter, van den Brink, Veltman, & Goudriaan, 2012; Yip et al., 2018).

As well as the distinct clinical features in different addictive behaviors, important confounding factors such as gender, comorbidity, and medication can no doubt contribute to the inconsistency. Moreover, the lack of statistical power is also a major problem, resulting from the typically small sample size in single study. In this setting, meta-analysis can be helpful. While a multi-modal meta-analysis including electroencephalography, magnetoencephalography, and functional MRI (fMRI) have detected common cue-reactivity activation across different BAs (Starcke, Antons, Trotzke, & Brand, 2018), structural studies have not yet been similarly integrated. Stable GM deficits have recently been reported in a meta-analytic way, but with only 10 studies on IGD, limiting the usefulness of its conclusion (Yao et al., 2017).

We therefore performed a large-scale voxel-wise meta-analysis (Radua et al., 2014) on diverse BAs including IGD, PG and other minorities (information and communication technologies addiction, food addiction, exercise addiction, shopping addiction, sexual addiction and work addiction). The aims were: (1) to discover robust brain structural differences between individuals with BAs and healthy controls. (2) To perform subgroup analysis to define the influence of confounding factors and the heterogeneity of these findings. (3) To conduct a meta-regression exploring the association between some addiction-related variants and GM alterations. We hypothesized that individuals with various forms of BAs would have shared structural abnormalities primarily in the prefrontal and striatal areas.

## Methods

### Selection of studies for meta-analysis

We carried out a comprehensive and exhaustive search in PubMed, Web of Science, Cochrane Library and ScienceDirect for publications from January 2000 up to January 2019. The search terms were: “behavioral addiction”, “internet addiction”, “internet gaming disorder”, “social media addiction”, “video game addiction”, “mobile phone dependence”, “internet communication addiction”, “pathological gambling”, “gambling disorder”, “compulsive buying”, “shopping addiction”, “workaholism”, “exercise addiction”, “sexual addiction”, “problematic hypersexual behavior”, “food addiction”, “eating addiction” coupled with “VBM”, “gray matter”, “voxel based morphometry”, “voxel-wise”. The reference lists of studies found and some high-quality reviews were investigated manually.

Studies were eligible if they met the following criteria: (1) diagnoses of BAs in each study were based on DSM, quantitative assessment tools or both; (2) VBM results were derived from comparison between individuals with BAs and healthy controls (HCs); (3) whole-brain analysis was conducted with peak coordinates in Talairach or Montreal Neurological Institute (MNI) space. Studies were excluded if (1) they did not use VBM; (2) peak coordinates were not reported, and not obtainable by contacting the corresponding authors; (3) only region of interest results were available; (4) datasets were partially duplicated among several publications (if so, studies with the larger sample size were included and the other(s) discarded); (5) inconsistent thresholds were applied in different regions. When studies divided individuals into three or more groups for comparison, datasets without comorbidities or medications were preferred. Our study conformed to the Preferred Reporting Items for Systematic Reviews and Meta-Analysis (PRISMA) guidelines (Liberati et al., 2009).

Two authors screened the included studies independently in order to obtain the following data: number of individuals in each group; gender ratio; mean age; diagnosis and diagnostic criteria; peak coordinates of abnormal brain region; duration of illness; severity; Barratt Impulsiveness Scale-11 (BIS-11) scores; other relevant technical and statistical information. Any divergence was discussed and settled by consensus.

### Meta-analysis of included studies

Recently, a popular software called Anisotropic effect-size signed differential mapping (AES-SDM) has been widely applied for meta-analytic neuroimaging works. In fact, AES-SDM is a statistical technique that can automatically reconstruct statistical parametric maps with previously reported peak coordinates and effect sizes, allowing subsequent statistical analyses to get various meta-outcomes. By combining original statistical parametric maps and peak coordinates, AES-SDM gives users an alternative when datasets comprise both maps and coordinates. By applying AES-SDM in neuroimaging meta-analysis, no voxel can appear to be simultaneously positive and negative since all the coordinates will be reconstructed in one map (Radua & Mataix-Cols, 2009). The use of anisotropic kernels provides more precise effect sizes for voxels and improves the robustness of the reconstructed maps even in the absence of full width at half maximum (Radua et al., 2014). Therefore, AES-SDM was used for our voxel-wise meta-analysis, investigating GM differences between individuals with BAs and HCs (Radua et al., 2014).

We synthesized relevant data extracted from each included study. Brief steps were as follows: (1) the *P* value or *z* value in some studies were converted into t value online (https://www.sdmproject.com/utilities/?show=Statistics) if no t value; (2) the effect-size brain maps of GM differences from each study were recreated respectively; (3) pooled analysis was conducted by means of a random effects model, weighted by sample size, variance and between-study heterogeneity. Here, the between-study heterogeneity that AES-SDM requires included image analysis software, stereotactic space of the reported coordinates and threshold type (corrected or uncorrected). Consistent with previous meta-analysis, voxel *P* < 0.005 was used as a significant threshold. Cluster extent threshold >10 voxels and peak height threshold >1 were determined to avoid false positive results (Radua & Mataix-Cols, 2009).

### Reliability, subgroup and meta-regression analysis

Between-study heterogeneity was examined to find the heterogeneous brain regions with Q statistics using a random effects model under the same threshold as before. To verify the stability and reliability of the findings, we carried out jackknife sensitivity analysis by discarding each dataset in sequence and repeating the pooled analysis with the rest. If certain brain region remains significant in most of the repeats, we can infer that the abnormality is replicable. To examine potential confounding factors, individuals were divided into four different subtypes for further subgroup analysis: IGD subjects, PG subjects, male participants and individuals without current psychotropic medication.

Finally, meta-regression analysis was performed to explore the association between GM alterations and clinical features including BIS-11 score, duration of illness and addiction severity. Based on the evidence that variables for meta-regression reported in less than nine studies might increase false positive rate (Radua & Mataix-Cols, 2009), only BIS-11 but no other clinical assessments could be studied. Moreover, BIS-11 assesses core impulsive trait in BAs, which is more typical to be a regressor than other scales assessing comorbid status, such as Beck Depression Inventory and Self-Rating Anxiety Scale. This meta-regression analysis could only be regarded as exploratory, with a more conservative threshold (*P *< 0.0005) to avoid false-positive findings (Radua & Mataix-Cols, 2009). As the studies used a variety of severity assessment scales and scoring methods, we applied the Percent of Maximum Possible (POMP) score, which can express the real severity level according to the possible minimum and maximum scores (Rogers & De Brito, 2016). This standardized measure is better than other standardization (e.g., *z* score), for which it permits comparison across studies and samples. To avoid potential bias of inaccurate assessment, we did this only for 11 studies that applied Likert scales, not those estimating severity with Y/N questionnaires. Publication bias was assessed by visual inspection of funnel plots constructed using AES-SDM, and quantified by Egger's test (Egger, Davey Smith, Schneider, & Minder, 1997).

## Results

### Enrolled studies and sample features

**Figure 1.**
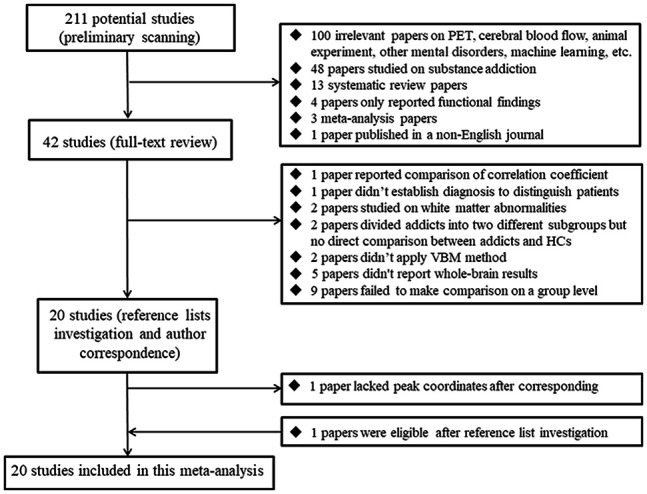
Procedure for including eligible studies in the meta-analysis. *Abbreviations*: HCs, healthy controls; PET, positron emission tomography; ROI, region of interest; VBM, voxel-based morphometry

The search in various databases identified 211 potential studies, of which 20 studies were eligible for meta-analysis comprising 505 individuals with BAs (451 males) and 564 HCs (514 males) (Fig. 1). Of these 20 studies, ten were of IGD (Choi et al., 2017; D. H. Han, Lyoo, & Renshaw, 2012; Jin et al., 2016; Ko et al., 2015; Lee, Namkoong, Lee, & Jung, 2018; Lin, Dong, Wang, & Du, 2015; Seok & Sohn, 2018a; Sun et al., 2014; Weng et al., 2013; Yoon et al., 2017), six of PG (Joutsa, Saunavaara, Parkkola, Niemela, & Kaasinen, 2011; Koehler et al., 2015; Mohammadi et al., 2016; van Holst, de Ruiter, et al., 2012; Yip et al., 2018; Zois et al., 2017), while the remaining four studies were of internet addiction, problematic hypersexual behavior, and mobile phone dependence (Seok & Sohn, 2018b; Y. Wang et al., 2016; Yuan et al., 2011; Y. Zhou et al., 2011). Thirteen of 20 studies recruited only male participants and no studies recruited only females. Relevant demographic, clinical and other characteristics are shown in Table 1.

**Table 1.** Demographic, clinical and methodological characteristics in the included studies

Study	Patients		Controls		Clinical characteristics				
	Sample size (M/F)	Mean age (Years)	Sample size (M/F)	Mean age (Years)	Diagnosis	Diagnostic criteria	Illness duration (years)	Severity^*^ (POMP score)	BIS-11
Y. Zhou et al. (2011)	18(16/2)	17.2	15(13/2)	17.8	IA	Modified YDQ	NA	NA	NA
Yuan et al. (2011)	18(12/6)	19.4	18(12/6)	19.5	IA	Modified YDQ	2.9	NA	NA
D. H. Han et al. (2012)	20(20/0)	20.9	18(18/0)	20.9	IGD	IAT + playing time	4.9	76.5	61.5
Weng et al. (2013)	17(13/4)	16.3	17(15/2)	15.5	IGD	Modified YDQ	NA	58.2	68.9
Sun et al. (2014)	18(15/3)	20.0	21(18/3)	22.0	IGD	Modified YDQ	NA	70.1	63.9
Ko et al. (2015)	30(30/0)	23.6	30(30/0)	24.2	IGD	DCIA	NA	82.1	78.5
Lin et al. (2015)	35(35/0)	22.2	36(36/0)	22.3	IGD	IAT + playing time	NA	65.0	NA
Jin et al. (2016)	25(16/9)	19.1	21(14/7)	18.8	IGD	IAT + DSM-V	6.0	54.1	NA
Choi et al. (2017)	22(22/0)	29.5	24(24/0)	27.2	IGD	DSM-V	NA	NA	NA
Lee et al. (2018)	31(31/0)	24.0	30(30/0)	23.0	IGD	IAT + DSM-V	9.9	55.5	54.4
Yoon et al. (2017)	19(19/0)	22.9	25(25/0)	25.4	IGD	IAT + playing time	6.3	70.4	70.1
Seok and Sohn (2018a)	20(20/0)	21.7	20(20/0)	22.4	IGD	DSM-V	NA	64.8	56.0
Joutsa et al. (2011)	12(12/0)	30.0	12(12/0)	27.0	PG	DSM-IV	NA	NA	NA
van Holst, de Ruiter, et al. (2012)	40(40/0)	36.5	54(54/0)	35.3	PG	DSM-IV-TR	12.2	NA	NA
Koehler et al. (2015)	20(20/0)	33.7	21(21/0)	39.2	PG	KFG	NA	NA	NA
Mohammadi et al. (2016)	15(15/0)	36.7	15(15/0)	36.8	PG	KFG	NA	NA	NA
Zois et al. (2017)	60(60/0)	36.7	98(98/0)	36.1	PG	DSM-IV	11.2	NA	NA
Yip et al. (2018)	35(26/9)	38.4	37(28/9)	38.0	PG	DSM-IV	NA	NA	70.1
Seok and Sohn (2018b)	16(16/0)	26.9	18(18/0)	25.1	PHB	SAST + HBI	10.6	58.9	52.5
Y. Wang et al. (2016)	34(13/21)	21.6	34(13/21)	21.7	MPD	MPAI	4.8	59.1	47.5

*Abbreviations*: BIS-11, Barratt Impulsiveness Scale-11; DCIA, Diagnostic Criteria of Internet Addiction; DSM, Diagnostic and Statistical Manual of Mental Disorders; HBI, Hypersexual Behavior Inventory; IAT, Internet Addiction Test; IGD, Internet gaming disorder; KFG, “Kurzfrageboge zum Glücksspielverhalten” (German gambling questionnaire); MPAI, Mobile Phone Addiction Index; MPD, mobile phone dependence; NA, not available; PG, pathological gambling; PHB, problematic hypersexual behavior; POMP, percent of maximum possible; SAST, Sexual Addiction Screening Test; YDQ, Young Diagnostic Questionnaire.

^*^POMP score = (raw score – possible minimum score) / (possible maximum score – possible minimum score) × 100.

### Regional GM differences and reliability analysis

**Figure 2.**
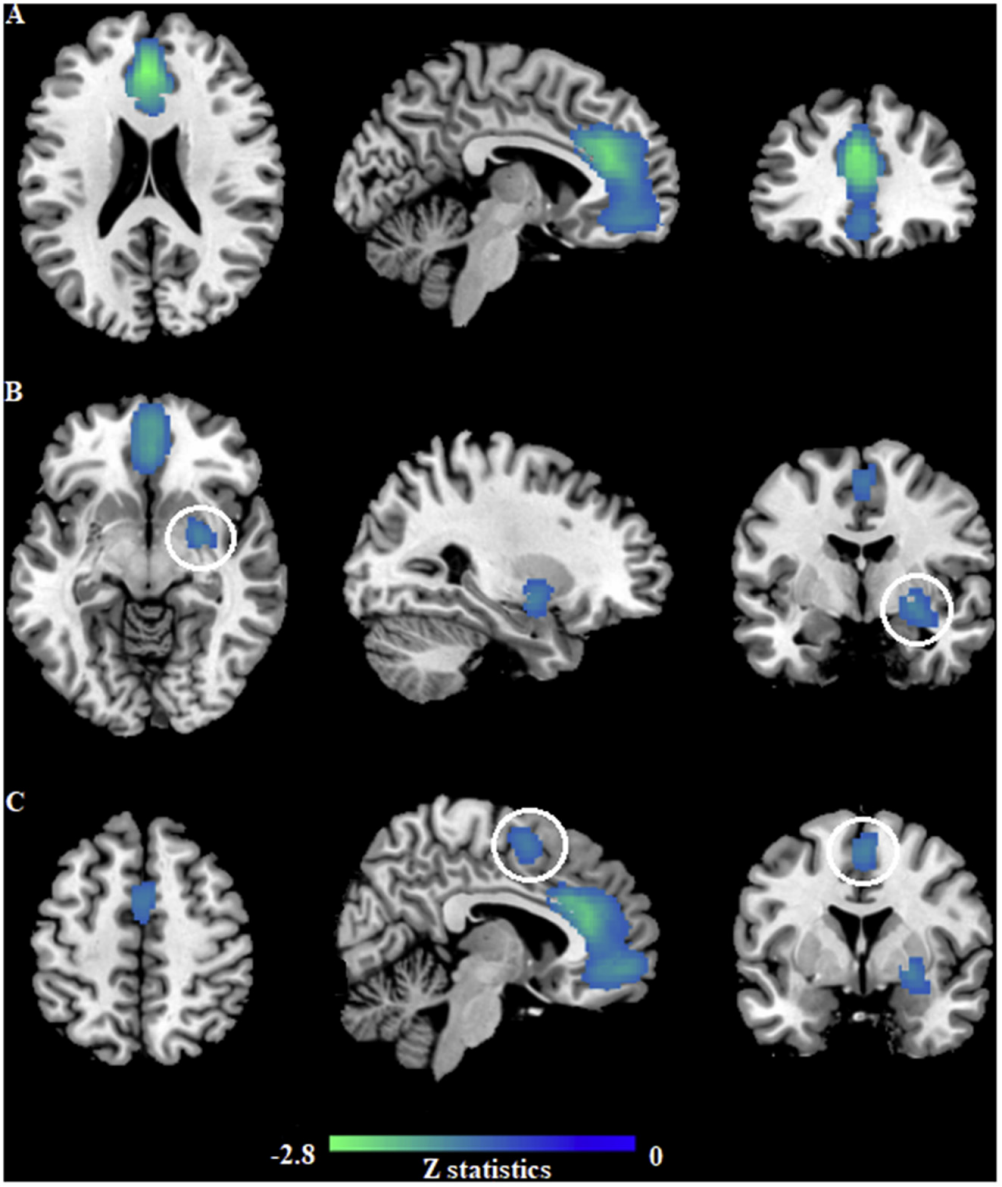
GM reductions for 505 individuals with BAs compared with 564 HCs. Clusters were shown in the sagittal, axial and coronal planes and the selected cluster was highlighted with a circle. Regions with GM enlargement were shown in red and GM reductions were displayed in blue. (A) GM reduction in the left ACC; (B) GM reduction in the right striatum; (C) GM reduction in the right SMA. *Abbreviations*: ACC, anterior cingulate cortex; BAs, behavioral addictions; GM, gray matter; HCs, healthy controls; MNI, Montreal Neurological Institute; SMA, supplementary motor area

As a result of pooled meta-analysis, the individuals with BAs (mainly IGD and PG) showed significant GM decreases in the left ACC extending to the left medial superior frontal gyrus (mSFG) and bilateral orbitofrontal gyrus (OFG), right putamen and right SMA. No brain regions showed significant GM increases (Table 2, Fig. 2).

**Table 2.** Regional GM volume differences between individuals with behavioral addiction and health controls in the main meta-analysis

Region	MNI coordinate (*x*, *y*, *z*)	SDM-Z value	*P* value	No. of voxels	Breakdown (No. of voxels)
*BAs < HCs*					
L anterior cingulate	−2, 38, 20	−2.827	<0.000001	3821	L anterior cingulate (1055)
					L medial superior frontal gyrus (650)
					R anterior cingulate (615)
					R medial orbitofrontal gyrus (408)
					L medial orbitofrontal gyrus (388)
					R median cingulate (162)
					R medial superior frontal gyrus (155)
					L median cingulate (127)
					L rectus (105)
					R rectus (51)
					Others (105)
R supplementary motor area	4, 2, 56	−1.694	0.000578	421	R supplementary motor area (229)
					L supplementary motor area (176)
					Others (16)
R putamen	28, −4, −10	−1.724	0.000475	337	R putamen (105)
					R amygdala (67)
					R pallidum (54)
					Others (111)

*Abbreviations*: BAs, behavioral addictions; HCs, healthy controls; GM, gray matter; MNI, Montreal Neurological Institute; SDM, signed differential mapping; L, left; R, right.

For the pooled results, there was regional inter-study heterogeneity (*P* < 0.05) in the right ACC and right middle frontal gyrus. In jackknife analysis discarding one of the 20 datasets at a time, GM decrease in the left ACC (20/20) was always reproducible, while GM decrease in the right striatum (19/20) and right SMA (19/20) survived in most of the repeated procedures. When discarding the studies of Lin et al. (2015) and Lee et al. (2018), the repetition failed respectively for the striatum and SMA (Table 3). Neither the funnel plot nor Egger's test showed significant publication bias (*P* > 0.05).

**Table 3.** Jackknife sensitivity of pooled meta-analysis

Removed study	L ACC	R striatum	R SMA
Y. Zhou et al. (2011)	Y	Y	Y
Yuan et al. (2011)	Y	Y	Y
D. H. Han et al. (2012)	Y	Y	Y
Weng et al. (2013)	Y	Y	Y
Sun et al. (2014)	Y	Y	Y
Ko et al. (2015)	Y	Y	Y
Lin et al. (2015)	Y	***N***	Y
Jin et al. (2016)	Y	Y	Y
Choi et al. (2017)	Y	Y	Y
Lee et al. (2018)	Y	Y	***N***
Yoon et al. (2017)	Y	Y	Y
Seok and Sohn (2018a)	Y	Y	Y
Joutsa et al. (2011)	Y	Y	Y
van Holst, de Ruiter, et al. (2012)	Y	Y	Y
Koehler et al. (2015)	Y	Y	Y
Mohammadi et al. (2016)	Y	Y	Y
Zois et al. (2017)	Y	Y	Y
Yip et al. (2018)	Y	Y	Y
Seok and Sohn (2018b)	Y	Y	Y
Y. Wang et al. (2016)	Y	Y	Y
Total	20 Y of 20	19 Y of 20	19 Y of 20

*Abbreviations*: ACC, anterior cingulate cortex; SMA, supplementary motor area; N, No; Y, Yes; L, left; R, right.

### Subgroup analysis

**Figure 3.**
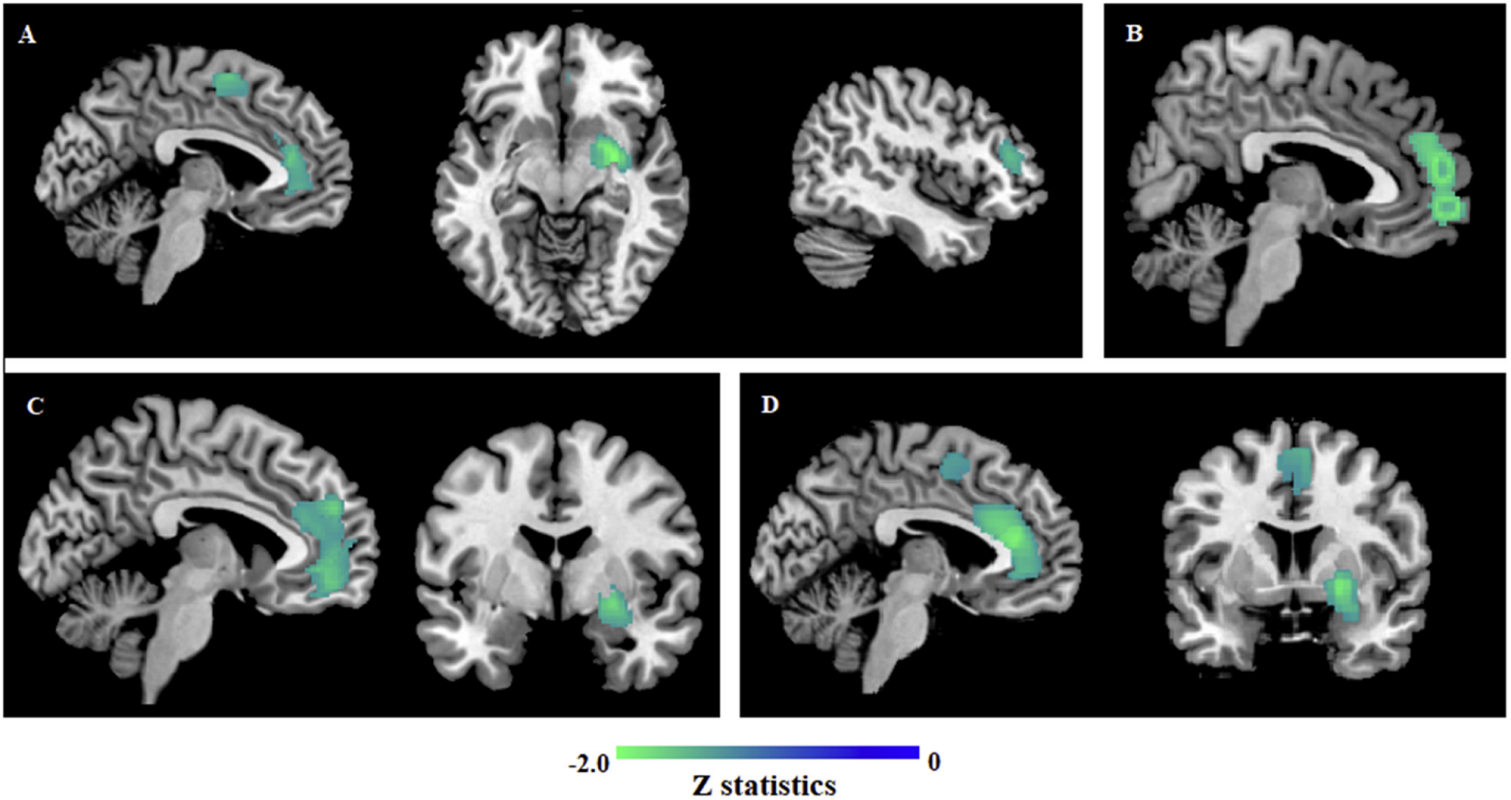
GM reductions for addicts from four specific subgroups compared with healthy controls. (A) Patients with IGD compared with HCs; (B) Patients with PG compared with HCs; (C) Male addicts compared with HCs; (D) Addicts without current psychotropic medication compared with HCs. Regions with GM enlargement were shown in red and GM reductions were displayed in blue. *Abbreviations*: GM, gray matter; HCs, healthy controls; IGD, internet gaming disorder; PG, pathological gambling

We performed subgroup analysis on different addictive behaviors to test the robustness of our pooled results. IGD and PG were able to form an independent subgroup respectively, while others failed because of insufficient studies. As shown in Table 4, findings in IGD (10 datasets) were substantially consistent with the pooled analysis, apart from the additional significant GM decrease in the right inferior frontal gyrus (Fig. 3A). Individuals with PG (six datasets) showed significant GM decrease in the left mSFG compared with HCs (Fig. 3B). Studies including only males were analyzed in order to investigate gender heterogeneity in BAs. As a result, male individuals with BAs compared with HCs (14 datasets) showed decreased GM volume in the left mSFG and right putamen (Fig. 3C). To rule out drug effects, studies excluding individuals who received psychotropic medications within 6 months prior to scanning (11 datasets) formed a medication-free subgroup. Significant GM decreases were shown in the left ACC, right putamen and right SMA, broadly as in the pooled analysis (Fig. 3D).

**Table 4.** Regional GM volume differences between individuals with behavioral addiction and healthy controls in the subgroup analyses

Region	MNI coordinate (*x*, *y*, *z*)	SDM-Z value	*P* value	No. of voxels	Breakdown (No. of voxels)
***Subgroup 1 (IGD, 10 datasets)***					
*IGD < HCs*					
R putamen	30, −2, −10	−2.105	0.000026	661	R putamen (176)
					R amygdala (136)
					R pallidum (70)
					R hippocampus (22)
					Others (257)
R supplementary motor area	4, 2, 62	−1.849	0.000330	554	R supplementary motor area (281)
					L supplementary motor area (228)
					Others (45)
L anterior cingulate	0, 38, 10	−1.861	0.000315	546	L anterior cingulate (313)
					R anterior cingulate (195)
					Others (38)
R inferior frontal gyrus, pars triangularis	46, 36, 18	−1.648	0.001331	165	R inferior frontal gyrus, pars triangularis (88)
					R middle frontal gyrus (77)
***Subgroup 2 (PG, 6 datasets)***					
*PG < HCs*					
L medial superior frontal gyrus	−2, 46, 26	−1.876	0.000036	1053	L medial superior frontal gyrus (526)
					R medial superior frontal gyrus (164)
					R medial orbitofrontal gyrus (105)
					L medial orbitofrontal gyrus (94)
					L anterior cingulate (54)
					Others (110)
***Subgroup 3 (male subjects, 13 datasets)***					
*BAs < HCs*					
L medial superior frontal gyrus	0, 44, 22	−2.059	0.000134	2299	L anterior cingulate (638)
					L medial superior frontal gyrus (482)
					R anterior cingulate (401)
					L medial orbitofrontal gyrus (256)
					R medial orbitofrontal gyrus (252)
					R medial superior frontal gyrus (136)
					Others (134)
R putamen	26, 0, −8	−1.772	0.000738	281	R putamen (72)
					R amygdala (68)
					R pallidum (48)
					Others (93)
***Subgroup 4 (medication-free subjects, 11 datasets)***					
*BAs < HCs*					
L anterior cingulate	0, 38, 12	−1.929	0.000057	1537	L anterior cingulate (748)
					R anterior cingulate (463)
					R median cingulate (117)
					L median cingulate (73)
					L medial superior frontal gyrus (43)
					Others (217)
R putamen	28, −2, −8	−1.925	0.000057	720	R putamen (176)
					R amygdala (170)
					R pallidum (93)
					R hippocampus (54)
					Others (227)
R supplementary motor area	6, 4, 60	−1.458	0.001388	443	R supplementary motor area (233)
					L supplementary motor area (162)
					Others (48)

*Abbreviations*: GM, gray matter; BAs, behavioral addictions; HCs, healthy controls; IGD, internet gaming disorder; PG, pathological gambling; L, left; R, right; MNI, Montreal Neurological Institute; SDM, signed differential mapping.

### Meta-regression

**Figure 4.**
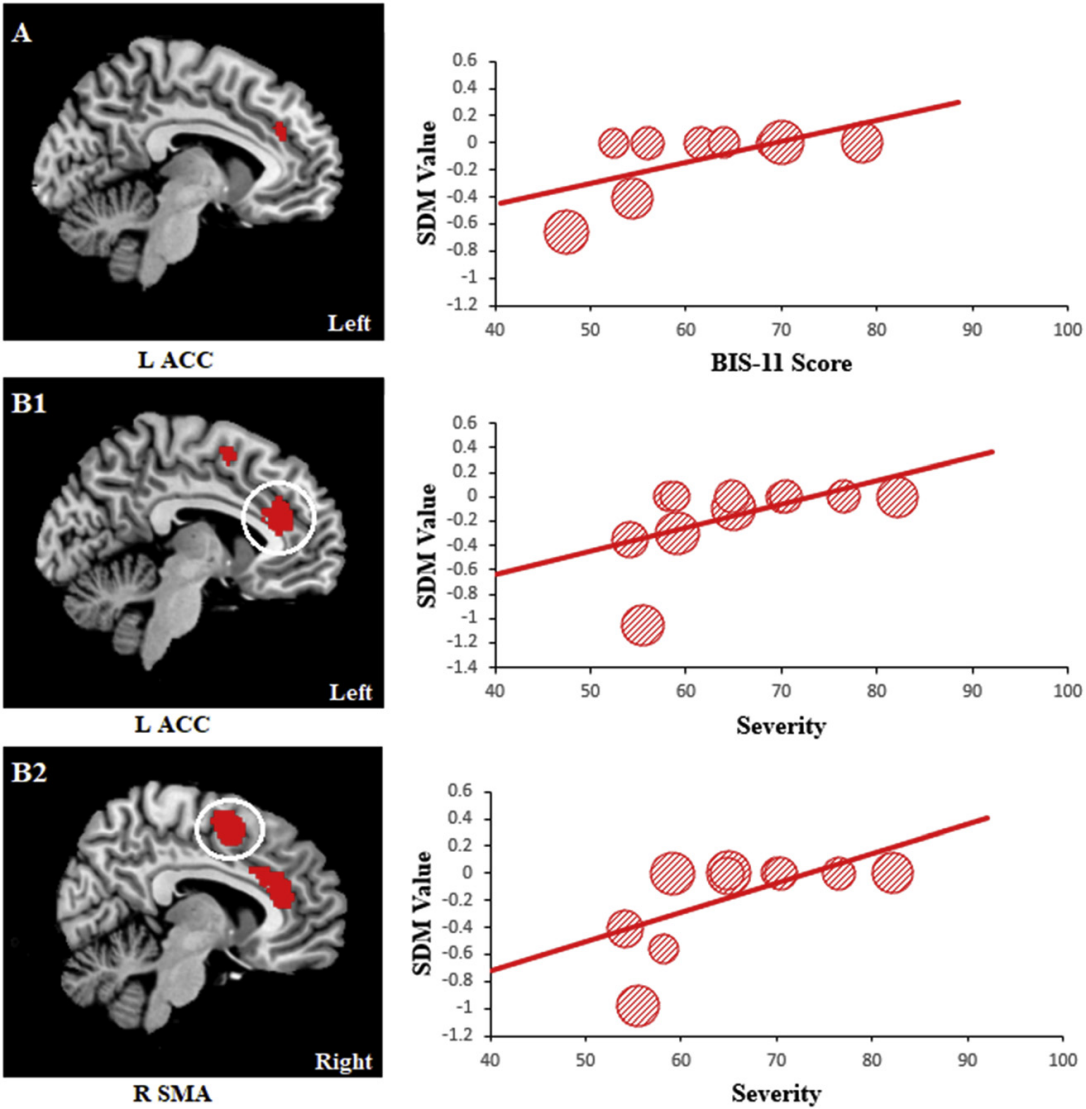
Results of the meta-regression analysis showing the correlation between several clinical features and regional GM reductions. (A) Positive correlation between BIS-11 scores and GM reduction in the left ACC; (B1) positive correlation between severity of BAs and GM reduction in the left ACC; (B2) positive correlation between severity of BAs and GM reduction in the right SMA. In this plot, each study is marked as a dot and the size of each dot depends on its sample size. The regression line (meta-regression signed differential mapping slope) is presented as a straight line. The SDM values (effect sizes) were extracted from the peak of maximum slope significance. Note the meta-regression SDM value is derived from the proportion of studies that reported gray matter changes near the voxel, so it is expected that some values are at 0 or near ±1. *Abbreviations*: ACC, anterior cingulate cortex; BAs, behavioral addictions; BIS-11, Barratt Impulsiveness Scale-11; GM, gray matter; L, left; R, right; SDM, signed differential mapping; SMA, supplementary motor area

Higher BIS-11 scores (10 datasets) in addicts were positively associated with GM reduction in the left ACC (MNI coordinate: −8, 36, 28; SDM-Z: 1.929; *P*: 0.00009; 51 voxels) (Fig. 4A). More severely affected individuals (11 datasets) had a higher GM reduction in the left ACC (MNI coordinate: −2, 36, 22; SDM-Z: 2.082; *P *< 0.00001; 871 voxels) (Fig. 4B1) and right SMA (MNI coordinate: 4, 2, 58; SDM-Z: 2.061; *P *< 0.00001; 556 voxels) (Fig. 4B2). No significant linear correlations were found with duration of illness.

## Discussion

To our knowledge, this is the first transdiagnostic meta-analysis investigating shared structural abnormalities in distinct BAs. Based on 20 VBM studies on five different kinds of BAs, shared GM decreases were observed in the left ACC extending to the left mSFG and bilateral OFG, right putamen and right SMA, stable and replicable under jackknife sensitivity analysis. This finding could become a preliminary implication of neural structural biomarker in BAs, though 16 studies were of IGD and PG. In subgroup analysis, IGD individuals showed the substantially same deficits as in the main analysis, while PG individuals showed decreased GM volume barely in the left mSFG. Studies included only male participants showed decreased GM volume in the left mSFG and right amygdala. BAs individuals without current psychotropic medication showed GM decrease in the left ACC, right putamen and right SMA, consistent with the main results. Meta-regression analysis found that GM decrease in the left ACC and right SMA had a positive correlation with the severity of addiction, and higher GM decrease in the left ACC was also associated with higher BIS-11 scores.

### Shared GM atrophy in individuals with BAs

Consistent with previous findings in substance addiction (Ersche, Williams, Robbins, & Bullmore, 2013), robust GM decrease in the prefrontal cortex (PFC) was observed in individuals with BAs as well. Given the dysfunction of PFC revealed in drug addiction (Goldstein & Volkow, 2011), it stands to reason that a similar pattern of PFC abnormalities may exist in BAs. Evidence from an integrated review has suggested deficits of cognitive control in PG can be ascribed to the aberrant activation of PFC (Moccia et al., 2017). Specifically, individuals with BAs have shown stronger activation of the ACC, OFC, and SFG in response to cue-related stimuli (Ko et al., 2009; Limbrick-Oldfield et al., 2017; Schulte, Yokum, Jahn, & Gearhardt, 2019). Impaired ACC activity can be detected in individuals with BAs during tasks probing decision making and response inhibition (van Holst, van Holstein, van den Brink, Veltman, & Goudriaan, 2012; Y. Wang et al., 2017), indicating the involvement of ACC in high-order executive functions. Moreover, metabolic evidence in the ACC has shown similar hypometabolism and abnormal metabolic connectivity in IGD (Kim et al., 2019). These convergent findings suggest that PFC abnormalities subserve the neurobiological underpinnings of impaired cognitive and executive functions associated with BAs development. Future prevention and intervention of BAs can therefore pay more attention to the pattern of PFC abnormalities, especially the ACC.

Individuals with BAs also demonstrated significant GM decrease in the right putamen compared with HCs. Consistent with our findings, striatal morphology analysis on PG has identified continuous structural alterations in the putamen along with the symptomatic deterioration. The striatum plays a critical part in processing inputs and outputs from numerous brain regions including prefrontal cortex, ventral tegmental area and thalamus (Yager, Garcia, Wunsch, & Ferguson, 2015). Decreased resting-state functional connectivity (FC) between the putamen and several PFC regions has been reported in individuals with BAs, and partial cortical-striatal FC was associated with addictive severity (Hong et al., 2015; Jin et al., 2016). In addition, studies have found cognitive behavior therapy can effectively normalize the aberrant prefrontal-striatal FC and ease the symptoms (X. Han et al., 2018), indicating prefrontal-striatal circuits may underlie both the pathological and therapeutic mechanism of BAs. From the perspective of neurophysiology, a positron emission tomography study has revealed increased dopamine synthesis in the putamen and even the entire striatum (van Holst et al., 2018). Therefore, the striatal abnormalities may damage the integrity of prefrontal-striatal circuit, resulting in enhanced dopamine synthesis and interfering with the regulation of reward system. On the other hand, an interesting finding that decreased GM volume in the dorsal striatum but not in the ventral striatum is enlightening. According to fMRI studies on substance addiction, the activation of ventral striatal reward system was associated with excessive drug use at an early stage while dorsal part dominated after the formation of habitual behaviors (Vollstadt-Klein et al., 2010; X. Zhou et al., 2019b), indicating the transition from heavy use to dependence is probably mediated by a functional ventral-dorsal shift. Therefore, the decreased GM volume in the dorsal striatum is consistent with such a functional shift theory, and the intervention strategies targeting ventral-dorsal shift may prevent high-risk individuals from both substance and behavioral addiction BAs.

GM atrophy in the SMA observed in our meta-analysis is a novel structural alteration different from substance addiction. Numerous studies on BAs have demonstrated structural and functional abnormalities in the SMA: smaller volume, thinner cortex, stronger connectivity, and higher amplitude of low frequency fluctuation (Lee, Park, Namkoong, Kim, & Jung, 2018; H. Wang et al., 2015; Yuan et al., 2013; Zhang et al., 2016). The SMA is critical for motor function, especially the voluntary action, corresponding to the process of response inhibition associated with addiction (Cunnington, Windischberger, Deecke, & Moser, 2003). Lower activity of the SMA/pre-SMA may underlie the poor regulation of voluntary action during a task probing response inhibition (Chen et al., 2015). Moreover, regression analysis has established a negative correlation between impulsiveness and GM volume in the SMA (Lee, Namkoong, et al., 2018). Given few studies have reported similar results in substance addiction, the development of behavioral impulsiveness may implicate a more complicated pathway than that of impulsive drug taking, and structural alterations in the SMA can be regarded as a unique biomarker for the discrimination between substance addictions and BAs.

Nevertheless, the causality between GM decrease and development of BAs is still inexplicable by means of the integration of cross-sectional studies. Endophenotype and prospective studies in substance addiction revealed that smaller volume in prefrontal and striatal areas may implicate the vulnerability for the addictive development (Becker et al., 2015; Ersche et al., 2013). Moreover, a study employing longitudinal design demonstrated GM volume in the OFC decreased along with the Internet gaming training, indicating a sequential alteration of brain structure during the development of IGD (F. Zhou et al., 2019a). Therefore, the structural alterations in BAs may be potentially regarded as a vulnerable factor in prodromal phase or a toxic effect in developing phase. In view of the uncertainty, trying to illustrate the neural mechanism of BAs with the structural differences is too early at present. Maybe it is more suitable to just take advantage of the sequential structural alterations to assess the severity and duration before the causality is figured out.

### Effects of subtype, gender and pharmacotherapy

Analyzing by subtypes of BAs, results of IGD were broadly consistent with the pooled analysis. Evidence from a recent multimodal meta-analysis has suggested similar fronto-striatal abnormalities in IGD (Yao et al., 2017), supporting the stability of our results. In contrast to IGD, results in the PG subgroup did not match the pooled results, with a significant GM decrease in the left mSFG. Reward type (monetary versus non-monetary rewards) has been implicated to cause subtle imbalance of brain activity in the prefrontal and striatal regions (Sescousse, Barbalat, Domenech, & Dreher, 2013). Therefore, abnormal subregions of the prefronto-striatal circuits may differ between individuals with PG and other BAs sensitive to monetary and non-monetary stimuli. However, the number of PG studies was low and individuals without comorbidities were difficult to recruit, which could lead to confounding effects (Ferguson, Coulson, & Barnett, 2011; Grant, Odlaug, & Schreiber, 2014). Inter-study clinical and methodological heterogeneity including comorbidity, medication use, severity, and threshold could also contribute to this discrepancy. No other addictive behaviors formed an analyzable subgroup because of the limited amount of included studies.

Gender heterogeneity was observed in the mSFG after integrating studies including pure male participants. The prevalence of some BAs, such as IGD, PG, and sexual addiction, is much higher in males than females (Wartberg, Kriston, & Thomasius, 2017). In addition, evidence from our study that 13 pure male datasets were substantially derived from IGD and PG indirectly supports the existence of gender heterogeneity. One structural study has reported similar results, with female IGD individuals showing decreased while males showing relatively increased thickness in the SFG when compared with same-sex recreational game users (Z. Wang et al., 2019). According to direct comparison of male and female IGD individuals, males have demonstrated significantly lower seed-based FC between the SFG and posterior cingulate cortex (Sun et al., 2019), implicating the association between disrupted default mode network and gender heterogeneity. Abnormalities in the SFG may therefore underlie the gender heterogeneity and gender vulnerability in BAs.

To eliminate possible pharmacotherapeutic effects, we excluded studies recruiting participants free of current psychotropic medication. The results were replicable despite the evidence that pharmacotherapy can alter GM volume in other psychiatric conditions (Sheline, Gado, & Kraemer, 2003; Vita, De Peri, Deste, Barlati, & Sacchetti, 2015). Notably, pharmacotherapy in BAs is usually used for controlling comorbid psychiatric disorders and improving unstable mood rather than targeting addiction directly (Nakayama, Mihara, & Higuchi, 2017). Moreover, lack of clarity on the confounding effects of psychosocial therapy status, severity of comorbidity, and type of comorbid psychiatric disorders make it premature to rule out structural effects of psychotropic medication in addicts. As robust our result seems to be, it should be interpreted with caution.

### Clinical association with addictive severity and impulsivity

Meta-regression analysis found that the severity of BAs was positively associated with GM decrease in the left ACC and right SMA, with smaller ACC and SMA in more severe individuals. Disrupted FC with these two regions has been found associated with severity ratings (Jin et al., 2016; van Holst, Chase, & Clark, 2014). Interestingly, more severely-affected PG individuals have higher levels of serotonin 1B receptors in the ACC (Potenza et al., 2013), providing supporting evidence from an alternative perspective. Dysfunction of both the ACC and SMA has been reportedly involved in the impaired response inhibition in BAs (van Holst and van Holstein, 2012; Chen et al., 2015), which is the core cognitive function of BAs. Thus, the symptomatic severity of BAs may be closely related to impaired response inhibition, which can be ascribed to the abnormalities in the ACC and SMA.

Meanwhile, we also found a positive association between impulsivity and GM decrease in the left ACC independent from the SMA. Such a relationship has been identified in a previous VBM study of IGD (Lee, Namkoong, et al., 2018). FC within the ACC was positively correlated to self-rated impulsiveness (Hinvest, Elliott, McKie, & Anderson, 2011), which might indicate a latent functional compensation for poor response inhibition resulting from the atrophy of the ACC. As an important dimension of symptomatic severity in BAs, though the impulsivity stems from the dysregulation of response inhibition, it may be more correlated with the ACC rather than the SMA.

### Implications and limitations

This meta-analysis throws new light on shared structural abnormalities in a broad group of BAs, with possible implications for both clinical interventions and future research. First, personalized therapeutic strategies could be recommended considering the sex, impulsivity, and medication status. Second, pharmacotherapy and physiotherapy targeted at the ACC and SMA might be effective, which will of course require multimodal validation and longitudinal investigation. Third, although our study excluded various disorders (e.g., trichotillomania, kleptomania, skin-picking disorder, pyromania) which are not yet regarded as BAs, they share substantial clinical features with BAs (e.g., impulsive control, emotional dysregulation and after-activity pleasure) (Grant, Brewer, & Potenza, 2006). As society develops, none can predict which novel behaviors that we have never heard about may become addictive in the future. Thus, it is nearly impossible for us to sort each behaviors and study all the BAs. The better way is to investigate the shared addictive-like symptoms rather than BA itself to further understand the neural mechanism underlying common manifestations.

However, we need to point out several limitations in our current study. First, there is not yet complete agreement on diagnostic criteria except IGD and PG. We therefore regarded PG and IGD as prototypical BAs, leading to limited studies on other types of BAs included in our meta-analysis. Second, results of subgroup analysis must be interpreted cautiously given the limited number of included studies. Publications with respect to PG are less than 10, failing to yield reliable results. Third, our investigation on gender difference is based on male individuals without a female group as a contrast. Though the result is consistent with previous studies, it is only exploratory. Fourth, inter-study heterogeneity in methodology (including software, thresholds, magnetic field strength) may influence our results, which can hardly be ruled out. Future research should focus on these aspects.

## Conclusions

In summary, our meta-analysis of BAs found shared and robust GM decreases in the left ACC, right striatum and right SMA, consistent with previous findings using other modalities. Evidence in such an integrated way was able to support the idea that frontal and striatal regions might serve as structural biomarkers of BAs, especially IGD and PG. Subgroup and meta-regression analysis further explored heterogeneity within BAs as well as association between clinical information and GM abnormalities, which could benefit clinical assessment and treatment. Future large-scale and longitudinal studies using multimodal methods would benefit our understanding of the similarities and differences among various BAs.

## Funding sources

This study was supported by the National Natural Science Foundation (Grant Nos. 81621003, 81820108018) and program for Changjiang Scholars and Innovative Research Team in University (PCSIRT, Grant No. IRT16R52) of China, and the Functional and Molecular Imaging Key Laboratory of Sichuan Province (FMIKLSP, Grant: 2019JDS0044).

## Authors' contribution

KQ analyzed the data and wrote the manuscript. KQ and FZ searched and screened the literature. FZ and TC designed the study. FZ, LL, WL and XS contributed to the interpretation of data. GJK, DL and QG contributed to the critical revision of the manuscript. All authors had full access to all the data in the study and take responsibility for the integrity of the data and the accuracy of the data analysis. KQ and FZ contributed to this work equally.

## Conflict of interest

The authors declared no potential conflicts of interest.
